# Transcriptome and Expression Profiling Analysis of the Hemocytes Reveals a Large Number of Immune-Related Genes in Mud Crab *Scylla paramamosain* during *Vibrio parahaemolyticus* Infection

**DOI:** 10.1371/journal.pone.0114500

**Published:** 2014-12-08

**Authors:** Chuping Xie, Yaping Chen, Wanwei Sun, Jun Ding, Lizhen Zhou, Shasha Wang, Shuqi Wang, Yueling Zhang, Dashi Zhu, Xiaobo Wen, Songnian Hu, Shengkang Li

**Affiliations:** 1 Guangdong provincial key laboratory of marine biology, Shantou University, Shantou, China; 2 Marine Biology Institute, Shantou University, Shantou, China; 3 The CAS Key Laboratory of Genome Sciences and Information, Beijing Institute of Genomics, Chinese Academy of Sciences, Beijing, China; Kansas State University, United States of America

## Abstract

**Background:**

Mud crab *Scylla paramamosain* is an economically important marine species in China. However, frequent outbreaks of infectious diseases caused by marine bacteria, such as *Vibrio parahaemolyticus*, result in great economic losses.

**Methodology/Principal Findings:**

Comparative *de novo* transcriptome analysis of *S. paramamosain* infected with *V. parahaemolyticus* was carried out to investigate the molecular mechanisms underlying the immune response to pathogenic bacteria by using the Illumina paired-end sequencing platform. A total of 52,934,042 clean reads from the hemocytes of *V. parahaemolyticus*-infected mud crabs and controls were obtained and assembled into 186,193 contigs. 59,120 unigenes were identified from 81,709 consensus sequences of mud crabs and 48,934 unigenes were matched proteins in the Nr or Swissprot databases. Among these, 10,566 unigenes belong to 3 categories of Gene Ontology, 25,349 to 30 categories of KEGG, and 15,191 to 25 categories of COG database, covering almost all functional categories. By using the Solexa/Illumina's DGE platform, 1213 differentially expressed genes (*P*<0.05), including 538 significantly up-regulated and 675 down-regulated, were detected in *V. parahaemolyticus*-infected crabs as compared to that in the controls. Transcript levels of randomly-chosen genes were further measured by quantitative real-time PCR to confirm the expression profiles. Many differentially expressed genes are involved in various immune processes, including stimulation of the Toll pathway, Immune Deficiency (IMD) pathway, Ras-regulated endocytosis, and proPO-activating system.

**Conclusions/Significance:**

Analysis of the expression profile of crabs under infection provides invaluable new data for biological research in *S. paramamosain*, such as the identification of novel genes in the hemocytes during *V. parahaemolyticus* infection. These results will facilitate our comprehensive understanding of the mechanisms involved in the immune response to bacterial infection and will be helpful for diseases prevention in crab aquaculture.

## Introduction

*Scylla paramamosain* (Crustacea: Decapods: Brachyura), an economically important mariculture commonly known as mud crab, is widely distributed in China and several parts of the world. However, recent rapid development of mud crab farming industry has led to increasingly severe outbreaks of infectious disease [1]. For example, the “milky disease” of mud crab, caused by pathogenic infection of bacteria *V. parahaemolyticus* in the last few years in Southern China, had resulted in more than 60% of mortality and thus brought in great economic losses [2]–[3].

Although much has been reported on the investigations of the innate immunity [4] and the characterization of immune-related genes in mud crab [5]–[9], little is known about the global molecular mechanisms underlying the immune response to such a pathogenic bacterial infection in this species. Due to a lack of genetic and genomic information, comprehensive gene expression analyses in the immune system have not been performed in mud crab. Cellular function of genes can be evaluated by the transcriptome analysis, which is a powerful method for assessing the relative importance of genes in cell, tissue, or organism under various conditions [10]. During the last few years, several methods, including ESTs sequencing [11]–[13], cDNA-AFLP [14]–[15], suppression subtractive hybridization (SSH) [16]–[17], and microarrays [18]–[19], have been applied to the study of transcriptome in maricultures. However, cDNA-AFLP and SSH have incomplete coverage [20], while EST sequencing techniques have limitations in the depth of transcriptome that can be sampled [21]. Microarrays are limited by background and cross hybridization problems. Further, microarrays measure the relative abundance of transcripts, and only predefined sequences can be detected [22].

When genome sequence is unavailable, transcriptome sequencing can be applied to decode the genomes of non-model organisms, providing valuable information to the understanding of gene function, cell responses and evolution [23]–[25]. High-throughput sequencing of RNA (RNA-Seq) has stimulated an unprecedented increase in transcriptome data [26]. The next-generation sequencing platforms, such as the Solexa/Illumina Genome Analyzer, ABI/SOLiD Gene Sequencer, and Roche/454 Pyrosequencer, can sequence in parallel massive amounts of DNA molecules derived directly from mRNA and produce millions or even billions of high-quality short reads [27]. The latest paired-end sequencing of RNA-Seq techniques have further improved the efficiency and expanded short read lengths, permitting a better understanding of the transcriptome data [28]. Recently, by using this technology, significant progress has been made in understanding the transcripts of various maricultures [29]–[32]. These results contribute greatly to the in-depth study of candidate genes in specific biological processes and provide rich sources for identification of novel genes. However, despite the economical importance of *S. paramamosain*, immunological investigation of the transcriptome has not been reported.

In this study, a transcriptome library of the hemocytes isolated from *V. parahaemolyticus*-infected crabs was constructed for massive parallel mRNA sequencing. Using the *de novo* transcriptome assembly software, we ultimately obtained a transcriptome database containing 81,709 unigenes. Quantitative gene expression analysis was performed using the Solexa/Illuminia's DGE technology, which identified 538 significantly up-regulated and 675 down-regulated genes from pathogen-infected crabs. A large number of the differentially expressed genes were immune-related and were further highlighted in signalling cascades.

## Results

### Transcriptome profile of mud crab *S.paramamosain*

**Figure 1:**
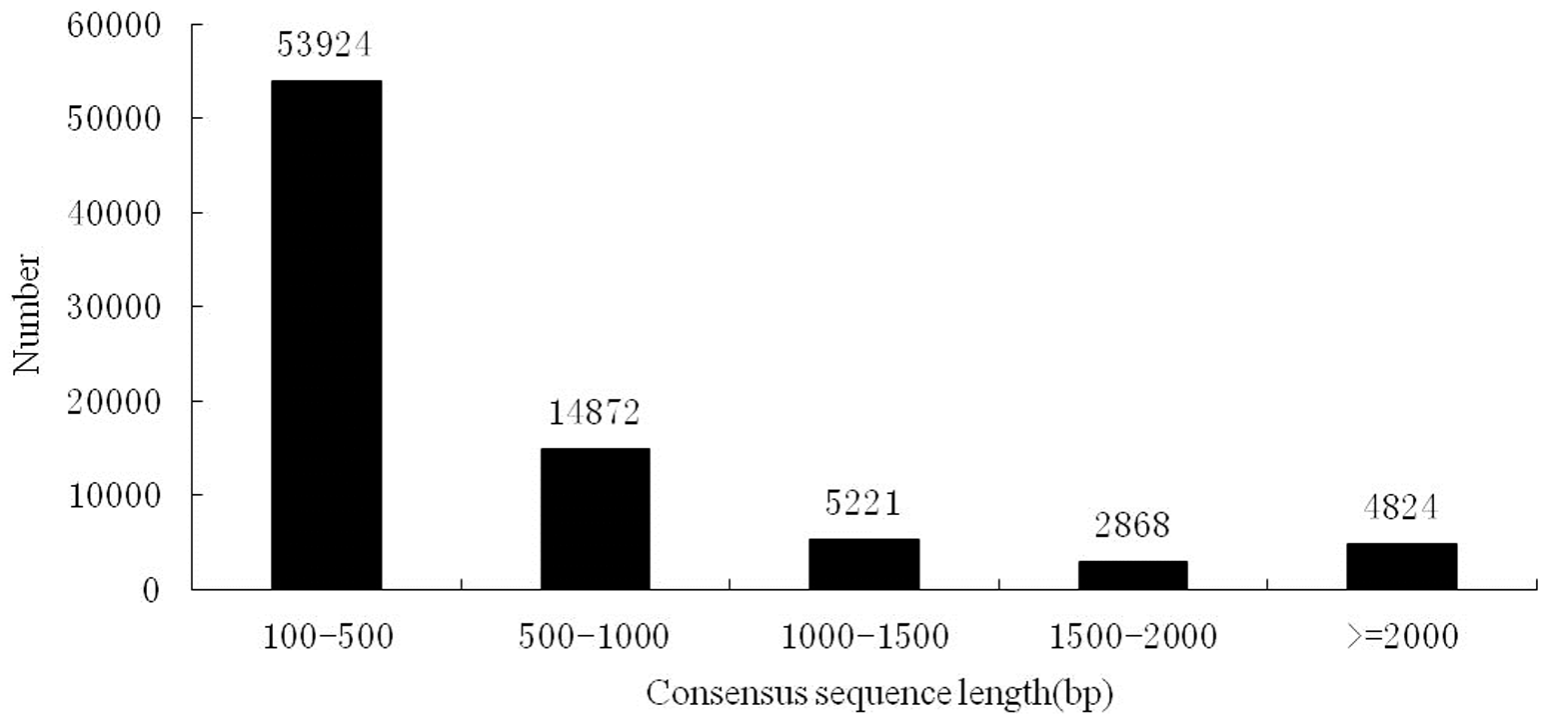
Length statistic of all consensus sequences obtained from the mud crab *S.paramamosain* transcriptome library.

Total RNA was extracted from the hemocytes of crabs at several time points, including 0, 12, 24, and 48 h after *V. parahaemolyticus* infection. Equal quantities of RNA were mixed together to construct a cDNA library for Illumina sequencing, which yielded a total of 62,004,374 reads. The reads containing more than five consecutive bases with a quality <13 were removed. The overall Illumina clean reads and bases are 52,934,042 and 4.76G, respectively. Files containing these data were deposited in the Sequence Read Archive of the National Center for Biotechnology Information (NCBI) with the accession number of SRP040563. The high-quality clean reads were assembled into 186,193 contigs by using the SOAP *de novo* software, which were further assembled into 81,709 consensus sequences with a mean length of 656 bp (range: 205∼23,885 bp). The length statistics of all consensus sequences is presented in Fig. 1.

59,120 unigenes were identified from 81,709 consensus sequences of mud crabs. After searching the Nr and Swissprot databases, 48,934 unigenes (31,482 in Nr and 27,498 in Swissprot) were identified. Among these, 10,566 unigenes belong to 3 categories of Gene Ontology, 25,349 to 30 categories of KEGG, and 15,191 to 25 categories of COG database, covering almost all functional categories (Table 1). The remaining 10,186 unigenes failed to match proteins in the Nr database or Swissprot databases and therefore represented potentially novel genes.

**Table 1:** Annotation of non-redundant unigenes.

Database	Number of annotated consensus sequences	Percentage of annotated consensus sequences
**Nr**	31,482	38.53%
**Swissprot**	27,498	33.65%
**Nt**	37,234	45.57%
**KEGG**	25,349	31.02%
**GO**	10,139	12.41%
**COG**	15,191	18.59%
**ALL**	59,120	

### GO, COG and KEGG Classification

**Figure 2:**
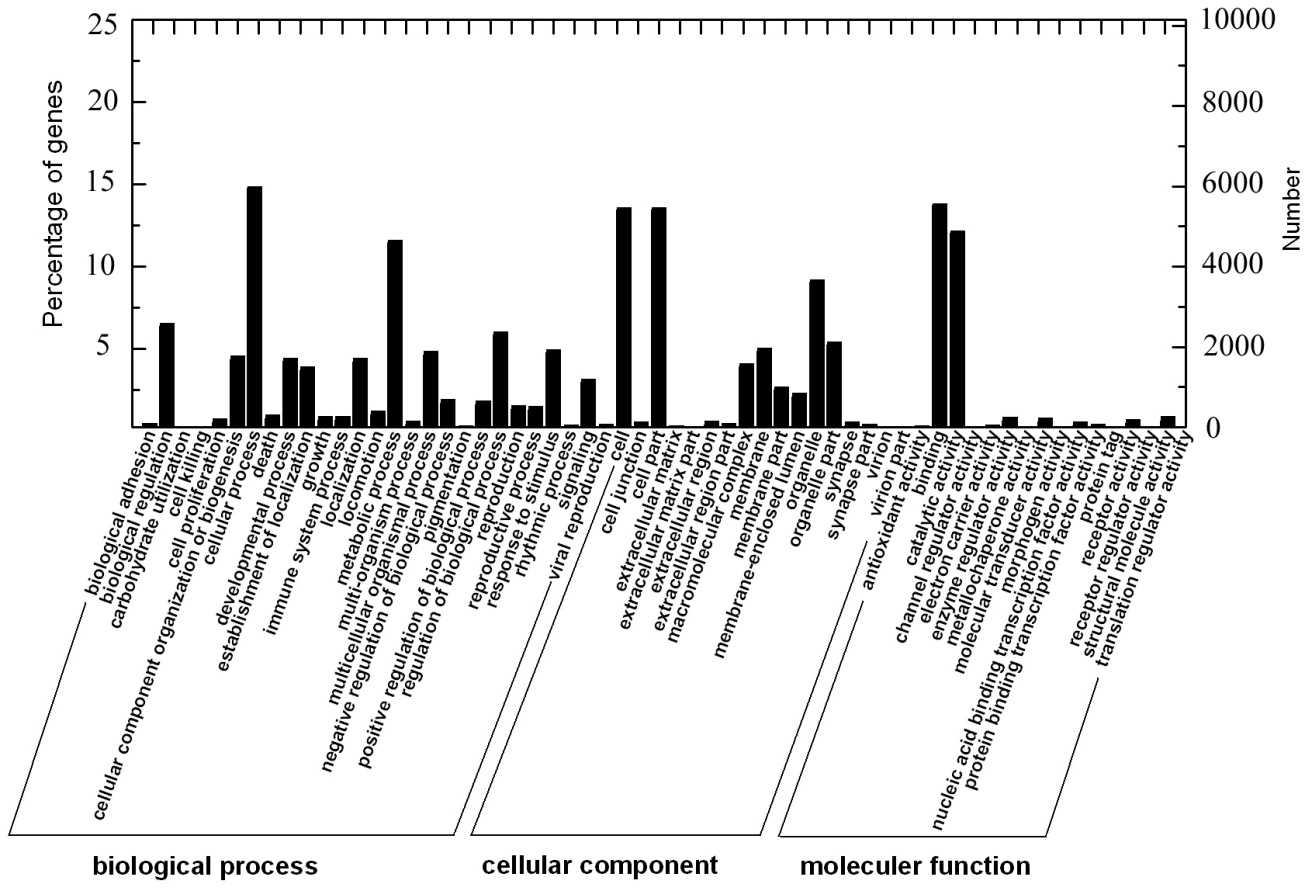
GO annotation of the unigenes in the *V.parahaemolyticus* infected crabs transcriptome. Most unigenes can be divided into three major categories, including biological process, cellular component, and molecular function.

Gene ontology (GO) analysis was performed using the web-based Database for Annotation, Visualization, and Integrated Discovery (DAVID) [32]–[33]. Among the 59,120 assembled unigenes, 10,566 of them were successfully annotated by GO assignments to one or more of the three categories: biological process, cellular component and molecular function (Fig. 2), which fell further into 27, 17 and 17 subcategories with the largest ones being the “primary metabolic process” (59.1%), “cell” (53.9%) and “binding” (55.0%), respectively. In summary, these terms account for a large fraction of the overall assignments in the *S. paramamosain* transcriptome data.

**Figure 3:**
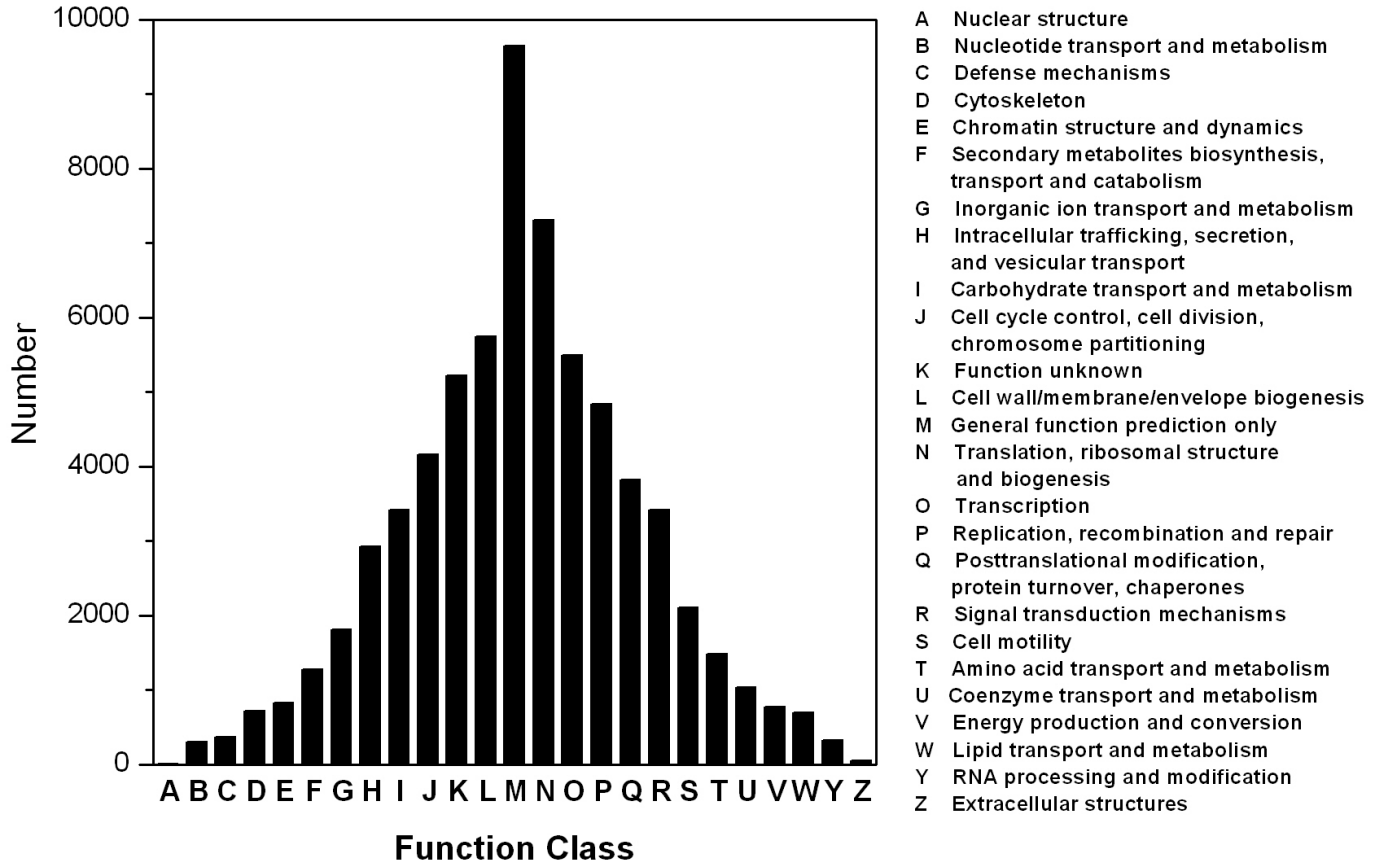
Histogram presentation of clusters of orthologous groups (COG) classification in *S.paramamosain*. All putative proteins were aligned to the COG database and could be classified into at least 25 molecular families.

Phylogenetic classifications of the predicted CDSs of unigenes were carried out by searching against the COG database (Fig. 3). A quantity of 15,191 unigenes were classified and subdivided into 25 COG categories (23 of them had known functions, while the other two were “general function prediction only” and “function unknown”), among which “General function prediction only” represented the largest group (9650 matched unigenes, 13.52%), followed by “Translation, ribosomal structure and biogenesis” (2,279, 14.08%) and “Cell wall/membrane/envelope biogenesis” (1,483, 9.16%).

A total of 25,349 unigenes in mud crab transcriptome were mapped to 30 statistically remarkable categories in KEGG (*P*<0.05) (S1 Table). Metabolic pathways (2701, 10.66%) and regulation of actin cytoskeleton (1610, 6.35%) were two of the most statistically significant categories. Notably, three immune related categories, mitogen-activated protein kinase (MAPK) signalling pathway (668, 2.64%), ubiquitin mediated proteolysis (594, 2.34%), and chemokine signalling pathway (761, 3.00%), were also identified as statistically significant.

### Global changes in gene expression upon *V.parahaemolyticus* infection

**Figure 4:**
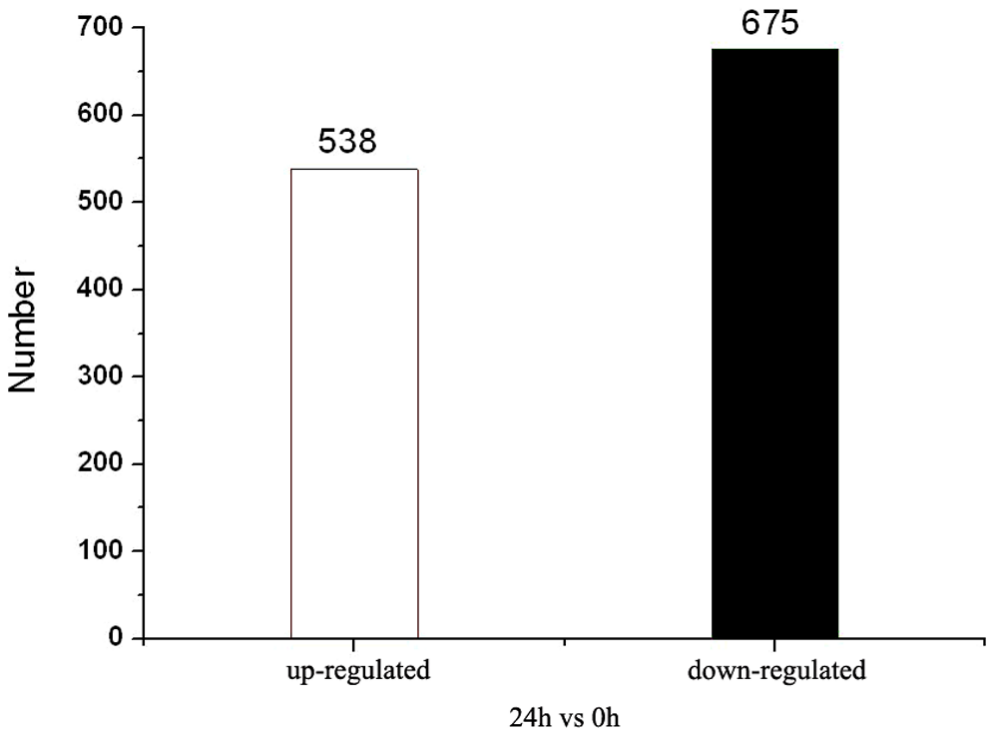
The differentially expressed genes upon *V.parahaemolyticus* infection. By using Solexa/Illumina's DGE platform, 1213 differentially expressed genes were detected in comparative analysis of the expression profiles between *V.parahaemolyticus*-infected crabs and control crabs, including 538 up-regulated genes and 675 down-regulated genes (*P*<0.05).

To characterize the immune response of mud crab to bacterial infection, two DGE libraries were constructed using mRNA from the haemocytes of crabs infected with *V.parahaemolyticus* and the controls, respectively. After removal of low-quality tags, adaptor tags, and one copy number tag, a total of 7,562,126 and 7,468,808 clean tags were obtained from the libraries of bacterium infected and control crabs, respectively (S2 Table), which then were mapped to the transcriptome database described above. A total of 1213 differentially expressed genes (*P*<0.05) were found, including 538 up-regulated and 675 down-regulated genes, in the haemocytes of crabs infected with *V.parahaemolyticus* (Fig. 4). Files containing these differentially expressed genes upon *V.parahaemolyticus* infection were deposited in the Gene Expression Omnibus (GEO) of NCBI with the accession number of GSE45400.

To achieve a functional annotation of the infection-responsive genes, GO classifications were assigned to the differentially expressed genes by using DAVID, indicating that the up- and down-regulated genes were involved in immunity, transcription, translation regulations, and biological regulation (S3 Table). Among the 1213 differentially expressed genes, 961 (79.23%) genes were well annotated, whereas the remaining 252 (20.77%) genes had low sequence homology to known sequences in public databases, suggesting that they might be putative novel genes in *S.paramamosain* involved in immune response to *V.parahaemolyticus* infection.

### Candidate genes involved in mud crab immune response

Of these 1213 differentially expressed genes, 96 of them were known to be involved in immunity and immune related signal transduction in mud crabs after *V. parahaemolyticus* infection (S4 Table). Most of these immune-related genes were enriched in GO terms “Protein metabolic” and “immune system related”, which contained up to 40 and 20 genes, respectively. The rest three categories were “cytokine and chemokine” (18 genes), “Transporter and regulator activity” (10 genes), and “Cell proliferation and apoptosis” (8 genes).

Genes involved in the Toll pathway and the Immune deficiency (IMD) pathway, such as Toll-like receptor (TLR), interferon (IFN) and tumor necrosis factor receptor 6 (TNFR6), were found significantly induced in mud crab upon *V. parahaemolyticus* infection. Antimicrobial proteins, such as Crustin, ALF2, and ALF6, were also significantly up-regulated, indicating they may play important roles in crab's defence against *V. parahaemolyticus* infection.

Genes annotated as Ras superfamily, including Ras, Rab and myosin, were also activated by *V. parahaemolyticus* infection. Many genes in the transcription regulation group and genes encoding cell proliferation and apoptosis, including Tob1 and Syntaxin, as well as genes involved in antioxidant activity, such as AST, CAT, CathepsinA and CathepsinD, were found up-regulated in crabs infected with *V. parahaemolyticus*.

Of these differentially expressed genes, a large number of immune related genes belong to the proPO-activating system, in which the most significantly differentially expressed genes were serine proteinases, clip domain serine proteinase, serine protease-like protein, chymotrypsin, and their inhibitors, such as serpin and pacifastin.

### Verification of transcriptome data by real-time RT-PCR

**Figure 5:**
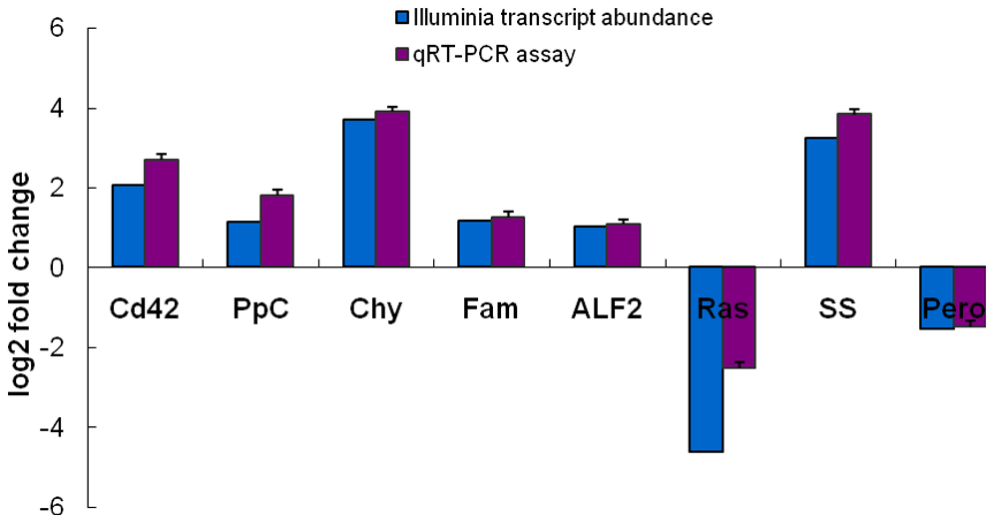
Comparison of the expression profiles of randomly selected genes as determinated by Illuminia sequencing and qRT-PCR analysis. Target gene abbreviations are as follows: Cd42, cell division cycle protein 42; PpC, phospholipid phospholipase C; Chy, chymotrypsin; Fam, farnesoic acid O-methyltransferase; ALF2, antilipopolysaccharide factor 2; Ras, Ras GTPase-activating protein; SS, son of sevenless; Pero, peroxinectin

Relative quantitative real-time RT-PCR (qRT-PCR) analysis was performed on randomly chosen genes (*Chy*, *Fam*, *ALF2*, *Ras*, *Cd42*, *PpC*, *SS*, *Pero*) to confirm the expression profiles of mud crabs in response to *V. parahaemolyticus* infection (Fig. 5). The results showed that qRT-PCR analyse of these genes was in good agreement with the Illuminia RNA-seq analyse, indicating the transcriptome data can reflect an actual gene expression profile in *V. parahaemolyticus* infected mud crabs.

## Discussion

*S. paramamosain* has received increasing attention over the years because of its wonderful flavor, and whose aquaculture production has reached 110,000 tons in China in 2011 [34]. *V. parahaemolyticus* infection is a main bacterial cause of crab mortality in cultured *S. paramamosain* in southern China during the last few years [2]. Although there are many reports on isolating and characterising immune-related genes in mud crabs, molecular studies on the immune response to pathogens in mud crab are still rare. To increase our knowledge of host responses to bacterial infection, we used Illuminia RNA-Seq to investigate the transcriptome profile of crab after *V. parahaemolyticus* infection, showing that a large proportion of unigenes from *S. paramamosain* were new transcripts compared to known genes in public databases by bioinformatics analysis. Comparative analysis of transcriptome changes between *V. parahaemolyticus*-infected and non-infected crabs revealed a large number of differentially expressed genes. Our sequencing data analyses indicate that *V. parahaemolyticus* infection has a significant impact on the transcriptional profile of *S. paramamosain* hemocytes. Previously, transcriptome data obtained from the 454 pyrosequencing platform have identified growth performance-related genes in mud crabs [34]. In another study, using the 454 GS FLX sequencing system, the authors identified 4,021 gonad-differentially, 10,522 ovary-specifically, and 19,013 testis-specifically expressed genes after comparing libraries of *S. paramamosain* [35]. Comparing our Illuminia RNA-Seq data with those from the 454 pyrosequencing platform, we found that the two sequencing platforms produced equivalent numbers of unigenes, however, a larger number of immune-related genes were identified in our study, while more growth-related and gonad-specific genes could be retrieved by the 454 pyrosequencing platform.

Among the differentially expressed genes found in this study, several, such as C-type lectin and catalase, have been previously reported to be involved in the host response against white spot syndrome virus (WSSV) and taura syndrome virus (TSV) [36]–[37]. In invertebrates, C-type lectins are involved in the non-self immune recognition and pathogen phagocytosis through opsonization [38]. It is indicated that catalase activity or increase in gene expression parallels immune-stimulant challenge or pathogen infections in maricultures [39]–[40]. In this study, we found several unigenes homologous to C-type lectins and 2 unigenes annotated as catalase, whose expression exhibited significant changes after *V. parahaemolyticus* infection. Similarly, heat shock protein, α2-macroglobulin, peroxinectin and alcohol dehydrogenase, have been reported to be strongly up-regulated in invertebrates when challenged with stressful conditions [7], [41]–[45]. In the present study, up-regulation of these genes has also been observed in crabs challenged with *V. parahaemolyticus*. Meanwhile, other significantly changed genes in mud crab during *V. parahaemolyticus* infection (such as calmodulin and integrin) have not been previously reported of roles in immune systems in aquatic animals. The immunological functions of these genes need further investigations.

The well-documented signaling pathways involved in the innate immune response of invertebrates against pathogens include the Toll pathway and immune deficiency (IMD) pathway, which can induce the expression of antimicrobial peptide (AMP) genes and regulate the host humoral response [30], [45]–[47]. The differentially expressed genes identified in crab's transcriptome data included Toll-like receptor (TLR), interferon (IFN) and tumor necrosis factor receptor 6 (TNFR6). Similar observations have been made in previous reports [30]. Further, many downstream effectors of Toll-IMD pathways, including *ALF2*, *ALF6*, crustins and other antimicrobial related peptides (S4 Table), were identified from the transcriptome data of crabs upon *V. parahaemolyticus* infection.

The Ras superfamily, a group of small GTPases exhibiting high-affinity binding for GDP and GTP, can be divided into several families based on their structures and functions [48]. By regulating actin and myosin recruitment, these GTPases are essential for phagocytosis [49]–[50]. Some subtypes of GTPases, such as Ras, Rab and myosin, were significantly induced (S4 Table), indicating that Ras-regulated phagocytosis was participated in the process of *V. parahaemolyticus* infection in mud crabs. Cell apoptosis plays significant roles in the control of immune response, the deletion of immune cells recognizing self-antigens, and cytotoxic killing [51]. Of these differentially expressed genes in mud crabs, genes involved in cell apoptosis/proliferation, including *Tab1*, *Cathepsin* D and *Syntaxin*, were activated by *V. parahaemolyticus* infection in mud crabs.

The proPO-activating system is composed of genes such as serine proteinases and their inhibitors (serpins), prophenoloxidase-activating enzyme (PPA), proPO and its active form, phenoloxidase (PO) [52]. In the present study, a number of differentially expressed genes found during *V. parahaemolyticus* infection in mud crabs were annotated to be tentative members of the proPO-activating system (S4 Table). These genes were mainly kinds of serine proteinases, including clip domain serine proteinase, serine protease-like protein, chymotrypsin, and their inhibitors, such as serpin and pacifastin. Hemocyanin is another well-known immune-related protein previously reported to possess multiple bioactive functions [53]. Hemocyanins have the defense-related functions that are mediated through phenoloxidase activity. Peroxinectin is present as a biologically inactive protein in the hemocytes in invertebrates and becomes converted into its active form, a cell adhesion molecule, when the proPO system is activated [52]. Hemocyanins, cryptocyanins and peroxinectin were found significantly induced upon *V. parahaemolyticus* infection in mud crabs. These data provided evidences that many genes in the proPO-activating cascade were stimulated by the pathogenic bacterium, indicating that the expression and regulation of different types of proPOs constituted a response to different kinds of pathogens in mud crabs. Last but not least, most of differentially expressed genes identified in the transcriptome of mud crabs in our study were annotated as unknown or hypothetical proteins, which would need more investigations in the future. Further identification of these genes and their functions might provide new insights into the immune response to *V. parahaemolyticus* infection.

In conclusion, the present study focused on the difference of the transcriptome of *V. parahaemolyticus*-infected and non-infected crabs, aiming at discovery of underlying mechanisms involved in host defense against pathogenic bacterium infection. Comparative transcriptome analysis of crabs of *V. parahaemolyticus*-infected and control groups showed significant differences in gene expression. Many immune-related genes as well as Toll and IMD pathway, Ras-regulated endocytosis, and proPO-activating genes, were affected by the infection and probably participated in antimicrobial process. In addition, this study provided a detailed data for identification of novel genes in the hemocytes in mud crab *S. paramamosain*, especially under the situation that the whole genome sequence of crab is not currently available.

## Methods

### Ethics statements

Mud crabs used in this study were taken under permission from a local crab farm (Niutianyang, Shantou, Guangdong, China) (23.3543^o^N. latitude and 116.6458^o^E. longitude). Crabs were processed according to “the Regulations for the Administration of Affairs Concerning Experimental Animals” established by the Guangdong Provincial Department of Science and Technology on the Use and Care of Animals.

### *S. paramamosain* sampling and the challenge experiment

Samples collection and the pathogen challenge had been described previously [54]. Briefly, forty healthy mud crabs (100±10 g in weight) were collected from Niutianyang area in Shantou, China. Crabs were acclimated for 3 days in 1 m^3^ tanks (ten crabs per tank) in conditions (salinity: 8‰; temperature: 28°C) similar to those of culture ponds from which crabs were obtained. Sea water was changed twice a day. Crabs were then fed with shellfishes for one week before sampling. *V. parahaemolyticus* isolated from diseased crabs (from Shantou crab farms) were cultured in 2216E medium at 28°C. 30 crabs were inoculated individually in the ventral hind legs with 100 µL of *V. parahaemolyticus* (2.4×10^7^ cells/mL), while the other 10 crabs were injected with 100 µL of saline (0.9%) as the control group. 10 crabs were harvested for haemocyte sampling at 12 h, 24 h, 48 h after the challenge. Haemocytes were collected from blood samples after mixing with anti-coagulant solution and centrifugation at 800 g at 4°C for 20 min. Haemocytes were immediately frozen in liquid nitrogen and then stored at −80°C prior to RNA extraction.

### RNA isolation

Total RNA was extracted from 50 to 100 mg of tissue with TRIZOL Reagent (Invitrogen, Carlsbad, CA) according to the manufacturer's instructions. The RNA samples were incubated for 30 min at 37°C with 10 units of DNase I (Takara, Dalian, China) to remove residual genomic DNA. The quality and quantity of the purified RNA were determined by measuring the absorbance at 260 nm/280 nm (A260/A280) using a ND-1000 spectrophotometer (LabTech, Holliston, MA) and integrity was ensured through analysis on a 1.5% (w/v) agarose gel.

### Library preparation and sequencing

cDNA libraries were constructed using RNA Sample Preparation Kit (Illumia, San Diego, CA) following manufacturer's instructions. Briefly, mRNA was purified from total RNA using polyATtract mRNA isolation systems (Promega, Madison, WI). Fragmentation was carried out using divalent cations under elevated temperature in Illumina proprietary fragmentation buffer. First strand cDNA was synthesized using random oligonucleotides and SuperScript II (Promega, Madison, WI). Second strand cDNA synthesis was subsequently performed using DNA Polymerase I and RNase H (Promega, Madison, WI). Remaining overhangs were converted into blunt ends via exonuclease/polymerase activities. In order to select cDNA fragments of preferentially 200 bp in length, the library fragments were purified with the AMPure XP system (Beckman Coulter, Beverly). Products were quantified using the Agilent high sensitivity DNA assay on the Agilent Bioanalyzer 2100 system. Finally, the library preparations were sequenced on an Illumina Hiseq 2000 platform which generates 100 bp paired-end reads.

### Assembly of transcripts and annotation

Raw sequencing reads were quality trimmed, and adaptor sequences were removed before the assembly. After removal of low quality reads, processed reads were assembled using SOAP *de novo* software [55] with default parameters. The overall assembly was performed using the combined sequence data for both treated crabs and the control groups. The contigs andsingletons were generally referred to as unigenes. As a result, 81,709 unigenes were generated. Subsequently, the unigenes were subjected to BLASTX similarity search against NCBI non-redundant protein database and the swissprot database using BLASTALL program with an E-value threshold of 10^−5^. All annotated unigenes were used to determine the COG term, GO term and KEGG pathway with a cut-off E-value of 10^−5^ using BLASTX [29]. The assembled contigs were used as a reference for annotating the DGE tags.

### Identification of differentially expressed genes

Gene expression was measured by counting tags from normal and pathogen-infected crabs and normalized to the total high-quality reads. High-throughput sequencing was performed using the Solexa/Illumina Genome Analyzer. The raw data was filtered to remove low quality sequences, including ambiguous nucleotides and adaptor sequences. The number of reads mapping to each unigene in the two populations analyzed (infected and non-infected) was counted and used as an approximate estimation of gene expression level in the corresponding individuals. For unigenes with multiple transcripts, the number of reads were summed and assumed to be the corresponding final read count. To link the expressed signatures to known genes from *crab*, the unigene dataset (http://trace.ncbi.nlm.nih.gov/Traces/sra_sub/sub.cgi?login=pda) from the transcriptome of *S. paramamosain* was used as a reference. The transcription level of each gene was deduced by determining the total number of reads mapped to each gene using Picard tools (http://picard.sourceforge.net/). To provide a relative assessment of transcript-abundance, the numbers of raw reads that were mapped to individual contigs were normalized for sequence length (i.e., Fragments Per Kilobase of transcript per Million mapped reads, FPKM). To investigate differences in gene expression profiles, FDR (false discovery rate) <0.001 was used as the threshold of *P*-value in multiple test to judge the significance of gene expression difference [56]. Genes were considered differentially expressed in a given library when the *P*-value <0.001 and a greater than two-fold change (absolute value of log2 ratio>1) in expression across libraries was observed.

### Quantitative real-time PCR

Quantitative real-time PCR was performed using the ABI Prism 7300 Detection System (Applied Biosystems, Foster City, CA) with SYBR Green I as the fluorescent dye according to the manufacturer's protocol (Takara, Dalian, China). First-strand cDNA was synthesized from 2 µg of total RNA as described above and used as a template for real-time PCR with specific primers (S5 Table). Real-time PCR was performed in a total volume of 20 µl, and cycling conditions were 95°C for 5 min, followed by 40 cycles of 94°C for 5 s, 60°C for 31 s. All reactions were performed in biological triplicates, and the results were expressed relative to the expression levels of β-actin in each sample by using the 2^-ΔΔCT^ method [57]. Each sample was first normalized for the amount of template added by comparison with the abundance of β-actin mRNA [5], [58].

### Statistical analysis

One way ANOVA analysis was used to analyze the means differences among samples. Pairwise t-test was then applied to compare gene expression levels between two crab populations. All statistical analyses (avo functions and pairwise t-test functions in R) were conducted in the R computation environment (http://www.r-project.org) [59]. *P*-values smaller than 0.05 were considered statistically significant.

## Supporting Information

S1 Table (XLS)

S2 Table (DOC)

S3 Table (XLS)

S4 Table (DOC)

S5 Table (XLS)
