# Supplementary material for: Transcriptome and Expression Profiling Analysis of the Hemocytes Reveals a Large Number of Immune-Related Genes in Mud Crab Scylla paramamosain during Vibrio parahaemolyticus Infection
Source: PLoS One. 2014 Dec 8;9(12):e114500. doi: 10.1371/journal.pone.0114500 (PMC4259333; doi:10.1371/journal.pone.0114500)
Supplement: S2 Table — (DOC) [file pone.0114500.s002.doc]

**Table S2, Output statistics of sequencing by DGE**

|  | **0h** | | | **24h** | |
| --- | --- | --- | --- | --- | --- |
|  | **number** | **percentage** | **number** | | **percentage** |
| **total clean reads** | 7468808 | 100.00% | 7562126 | | 100.00% |
| **total basepairs** | 365971592 | 100.00% | 370544174 | | 100.00% |
| **total mapped reads** | 6390802 | 85.57% | 6553100 | | 86.66% |
| **perfect match** | 5478422 | 73.35% | 5667083 | | 74.94% |
| **unique match** | 4837843 | 64.77% | 4909167 | | 64.92% |
| **total unmapped reads** | 1078006 | 14.43% | 1009026 | | 13.34% |
