# Supplementary material for: Transcriptome and Expression Profiling Analysis of the Hemocytes Reveals a Large Number of Immune-Related Genes in Mud Crab Scylla paramamosain during Vibrio parahaemolyticus Infection
Source: PLoS One. 2014 Dec 8;9(12):e114500. doi: 10.1371/journal.pone.0114500 (PMC4259333; doi:10.1371/journal.pone.0114500)
Supplement: S4 Table — (DOC) [file pone.0114500.s004.doc]

**Table S4,** Representatives of putative immune relevant genes/homologs as predicted by DGE

| **Catalogs** | **Unigene ID** | **Putaive Identification** | **log_2_ Ratio** | | | |
| --- | --- | --- | --- | --- | --- | --- |
| **Cell proliferation and cell apoptosis** | | | | | | |
| Tob | 10199 | Protein Tob1 | 2.23 | | |  |
|  | 1483 | selenophosphate synthetase | 1.24 | | |  |
| E1B | 24045 | adenovirus E1B 19 kDa protein-interacting protein | 1.98 | | |  |
| cathepsin | 1779 | cathepsin A | -1.14 | | |  |
|  | 5790 | cathepsin D | -1.52 | | |  |
| casein kinase | 1545 | casein kinase I isoform epsilon-like | 2.98 | | |  |
|  | 18260 | short gastrulation protein | 1.63 | | |  |
| syntaxin | 1205 | syntaxin | 1.25 | | |  |
| GATA | 4915 | GATA binding protein 5 | 1.23 | | |  |
| PERK | 1656 | type-I transmembrane ER-resident serine/threonine kinase PERK | 1.05 | | |  |
| **Transporter and regulator activity** | | | | | | |
|  | 10497 | sodium/hydrogen exchanger | 1.67 |  | | |
|  | 7683 | zinc transporter ZIP13-like | 1.22 |  | | |
|  | 2077 | sodium-dependent phosphate transporter | -1.03 |  | | |
| ATPase | 3718 | V-type proton ATPase subunit H isoform 1 | -1.04 |  | | |
|  | 1695 | Na+/K+/2Cl- cotransporter | -1.35 |  | | |
|  | 2203 | ornithine decarboxylase antizyme | -1.18 |  | | |
|  | 15763 | Disheveled-associated activator of morphogenesis | -1.19 |  | | |
|  | 41310 | multidrug resistance-associated protein member 2 | 4.50 |  | | |
|  | 2713 | phospholipid phospholipase C beta isoform | 1.14 |  | | |
|  | 2826 | regulator of g protein signaling | -1.44 |  | | |
| **Protein metabolic** | | | | | | |
|  | 25417 | acetyl-coenzyme A synthetase 2-like, mitochondrial-like | 4.02 | |  | |
|  | 16027 | trehalose 6-phosphate synthase 1 | 3.54 | |  | |
|  | 1897 | Multidrug resistance-associated protein 1 | 3.04 | |  | |
|  | 28281 | sepiapterin reductase-like | 2.95 | |  | |
|  | 15509 | multidrug resistance-associated protein 1-like | 2.85 | |  | |
|  | 7702 | matrix metalloproteinase | 2.85 | |  | |
|  | 15725 | acetyl-coenzyme A synthetase 2-like, mitochondrial-like | 2.56 | |  | |
|  |  |  |  | | | |
| MRCK | 3279 | serine/threonine-protein kinase MRCK beta | -1.22 | | | |
|  | 19522 | protein-L-isoaspartate O-methyltransferase, putative | -1.31 | | | |
|  | 13258 | Alpha-1,3-mannosyl-glycoprotein 4-beta-N-acetylglucosaminyltransferase B | -1.35 | | | |
|  | 7275 | isocitrate dehydrogenase | -1.69 | | | |
|  | 654 | folylpolyglutamate synthase, mitochondrial | 3.02 | | | |
|  | 9915 | Centromere protein V | 1.92 | | | |
|  | 13034 | tetratricopeptide repeat protein 1-like | 1.44 | | | |
|  | 3689 | tripartite motif-containing 32 | 1.04 | | | |
| H2A | 8598 | H2A histone family, member Y-like | 1.02 | | | |
| SMC4 | 21169 | SMC4, structural maintenance of chromosome protein 4 | -1.02 | | | |
|  | 2203 | ornithine decarboxylase antizyme | -1.18 | | | |
|  | 12687 | single-stranded DNA-binding protein 3-like isoform 2 | -1.22 | | | |
| U11/U12 | 13787 | small nuclear ribonucleoprotein 35 kDa protein-like | -1.95 | | | |
| **Immune system related** | | | | | | |
|  | 2377 | α2-macroglobulin-like | 2.97 | | | |
|  | 6440 | protein disulfide isomerase A6 | 1.47 | | | |
|  | 1129 | selenium-dependent glutathione peroxidase | 1.57 | | | |
| NUAK | 11719 | NUAK family SNF1-like kinase 1 | 1.33 | | | |
|  | 4377 | cysteine dioxygenase | 4.79 | |  | |
| CAD | 21417 | CAD protein-like | 2.07 | |  | |
|  | 3133 | capping protein (actin filament) muscle Z-line, alpha 1 | 1.92 | | | |
|  |  |  |  | | | |
|  | 3931 | glutamate synthase | 1.64 | | | |
| AMP | 3832 | AMP deaminase 2 isoform 2 | 1.58 | | | |
|  | 1483 | selenophosphate synthetase | 1.24 | | | |
|  | 13693 | 6-phosphofructo-2-kinase/fructose 2,6-bisphosphatase | 1.11 | | | |
|  | 7439 | ATP-binding cassette sub-family C member 9-like | 1.08 | | | |
|  | 6467 | ectonucleotide pyrophosphatase/phosphodiesterase family member 3 | -1.05 | | | |
|  | 13317 | acid alpha-glucosidase | -1.05 | | | |
|  | 9066 | RB13-6 antigen | -1.08 | | | |
|  | 6489 | lysine-specific demethylase lid isoform 2 | -1.13 | | | |
|  |  |  |  | | | |
|  | 5122 | hexaprenyldihydroxybenzoate methyltransferase | -1.15 | | | |
|  | 15763 | Disheveled-associated activator of morphogenesis | -1.19 | | | |
|  | 8093 | exocyst complex component 5-like | -1.04 | | | |
|  | 5189 | α2-macroglobulin | 3.18 | | | |
| CAT | 2728 | catalase | 1.92 | | | |
|  | 7702 | matrix metalloproteinase | 2.85 | | | |
| HSP | 4780 | Heat shock protein | 2.42 | | | |
|  | | | | | | |
| lectin | 31175 | C-type lectin 2 | 12.72 | | | |
|  | 17645 | calmodulin | 4.75 | | | |
|  | 1545 | calmodulin-like | 2.98 | | | |
| Integrin | 1869 | Integrin | 1.75 | | | |
| ALF | 1450 | antilipopolysaccharide factor 6 | 1.08 | | | |
|  | 1773 | antilipopolysaccharide factor 2 | 1.07 | | | |
| Ras | 2792 | ras-related protein Rab | 1.05 | | | |
|  | 4857 | myosin | 2.03 | | | |
|  | 848 | Ras GTPase-activating protein | -4.61 | | | |
| TLR | 3272 | Toll-like receptor | 1.32 | | | |
| IFN | 3491 | interferon | 1.93 | | | |
|  | 13230 | tumor necrosis factor receptor-associated factor 6 | 1.05 | | | |
|  | 2567 | clip domain serine proteinase | 2.35 | | | |
|  | 4419 | clip domain serine proteinase 3 | 1.71 | | | |
|  | 2097 | clip domain serine proteinase 2 | -1.11 | | | |
|  | 4330 | serine protease-like protein | 2.12 | | | |
|  | 1612 | peroxinectin | -1.55 | | | |
| Chy | 1852 | chymotrypsin | 1.72 | | | |
|  | 1129 | serpin | 1.57 | | | |
|  | 16836 | serine/threonine kinase | 1.52 | | | |
| HCS | 10418 | hemocyanins | 1.31 | | | |
